# Preoperative prediction of pathological grade in pancreatic ductal adenocarcinoma based on ^18^F-FDG PET/CT radiomics

**DOI:** 10.1186/s13550-021-00760-3

**Published:** 2021-02-25

**Authors:** Haiqun Xing, Zhixin Hao, Wenjia Zhu, Dehui Sun, Jie Ding, Hui Zhang, Yu Liu, Li Huo

**Affiliations:** 1 Department of Nuclear Medicine, Peking Union Medical College Hospital, Dongcheng District, Chinese Academy of Medical Science, Peking Union Medical College, No.1 Shuaifuyuan, Beijing, 100730 China; 2 Beijing Key Laboratory of Molecular Targeted Diagnosis and Therapy in Nuclear Medicine, Beijing, 100730 China; 3 Sinounion Healthcare Inc., Building 3-B, Zhongguancun Dong Sheng International Pioneer Park, Beijing, 100192 China; 4 Department of Biomedical Engineering, School of Medicine, Tsinghua University, Beijing, 100084 China

**Keywords:** ^18^F-FDG PET/CT, Pancreatic cancer, Radiomics, Machine learning, XGBoost

## Abstract

**Purpose:**

To develop and validate a machine learning model based on radiomic features derived from ^18^F-fluorodeoxyglucose (^18^F-FDG) positron emission tomography/computed tomography (PET/CT) images to preoperatively predict the pathological grade in patients with pancreatic ductal adenocarcinoma (PDAC).

**Methods:**

A total of 149 patients (83 men, 66 women, mean age 61 years old) with pathologically proven PDAC and a preoperative ^18^F-FDG PET/CT scan between May 2009 and January 2016 were included in this retrospective study. The cohort of patients was divided into two separate groups for the training (99 patients) and validation (50 patients) in chronological order. Radiomics features were extracted from PET/CT images using Pyradiomics implemented in Python, and the XGBoost algorithm was used to build a prediction model. Conventional PET parameters, including standardized uptake value, metabolic tumor volume, and total lesion glycolysis, were also measured. The quality of the proposed model was appraised by means of receiver operating characteristics (ROC) and areas under the ROC curve (AUC).

**Results:**

The prediction model based on a twelve-feature-combined radiomics signature could stratify PDAC patients into grade 1 and grade 2/3 groups with AUC of 0.994 in the training set and 0.921 in the validation set.

**Conclusion:**

The model developed is capable of predicting pathological differentiation grade of PDAC based on preoperative ^18^F-FDG PET/CT radiomics features.

## Introduction

Pancreatic ductal adenocarcinoma (PDAC) is the fourth leading cause of cancer-related death worldwide, which accounts for about 85% of all pancreatic tumors [1, 2]. Surgical resection is the only curative treatment for PDAC, and the addition of chemotherapy in the adjuvant setting has been shown to improve survival rates [3]. Despite the advances in diagnostic and therapeutic modalities, the prognosis of PDAC remains poor, with a reported 5-year survival rate of 7.2% [4]. Therefore, it is crucial to identify the prognostic factors that may predict the prognosis of patients with PDAC, which can help clinical physicians select patients for the available treatment options.

Pathological subtype is considered to be a crucial factor in PDAC prognosis, and the presence of poor differentiation portends a poor prognosis [5–7]. Intraoperative frozen sections are used for the patients undergoing pancreatectomy; however, this method is time-consuming and expensive relatively. For the patients with non-resectable tumor, endoscopic ultrasound-guided fine-needle aspiration (EUS-FNA) is a common approach to get the specimen of the tumor for pathological examination before making a treatment plan [8]. Nevertheless, this technique is highly invasive, which has the inherent risk of interventional complications, and the achievable samples are too limited to give a reliable histological grading [9]. The accuracy of EUS-FNA to determine the grade of tumor is still challenging in clinical practice [10, 11]. There is a need for a reliable technique to evaluate the differentiation of tumor before treatment.

Noninvasive imaging tools are playing a vital role in the preoperative evaluation of PDAC. Triphasic computed tomography (CT) is the best initial diagnostic method for PDAC, and magnetic resonance imaging (MRI) can be used in patients who cannot tolerate the intravenous contrast for CT [7]. Compared to traditional imaging methods, ^18^F-fluorodeoxyglucose (^18^F-FDG) positron emission tomography/computed tomography (PET/CT) can combine functional information and anatomic information. Over the past 20 years, studies have shown the potential of ^18^F-FDG PET/CT in the diagnosis, staging, and recurrence of PDAC [12]. ^18^F-FDG PET/CT parameters, including standardized uptake value (SUV), metabolic tumor volume (MTV), and total lesion glycolysis (TLG), have been reported as prognostic factors for pancreatic cancer [13–15]. However, established ^18^F-FDG PET/CT parameters are difficult to assess the histopathological differentiation of tumor [16]. The ability of PET/CT to noninvasively evaluate the differentiation of tumor has not been fully explored.

Radiomics, which was initially proposed by Lambin et al. in 2012 and defined as high-throughput extraction of large amounts of features from radiographic images, has the ability to capture intratumoral heterogeneity noninvasively [17, 18]. Radiomics has the potential to capture information beyond what is visible to the human eyes in an objective and repeatable way [19]. For the last decade, an increasing number of studies have been conducted to assess the efficacy of radiomics for the characterization of tumor heterogeneity [20]. Texture analysis can reflect the intratumoral heterogeneity to help assess tumor aggressiveness, and lower tumor heterogeneity is likely related to lower histological differentiation [21, 22]. Previous studies suggested that features derived from CT, MRI, and PET/CT favor the tumor classification or survival prediction [10, 23–26].

To our knowledge, there have been a few published reports regarding the value of PET/CT-based texture analysis for evaluating the histopathological differentiation of pancreatic tumor. Therefore, the purpose of our study was to develop a machine-learning model based on radiomic features extracted from PET/CT images for predicting the pathologic differentiation of PDAC preoperatively.

## Materials and methods

**Fig. 1:**
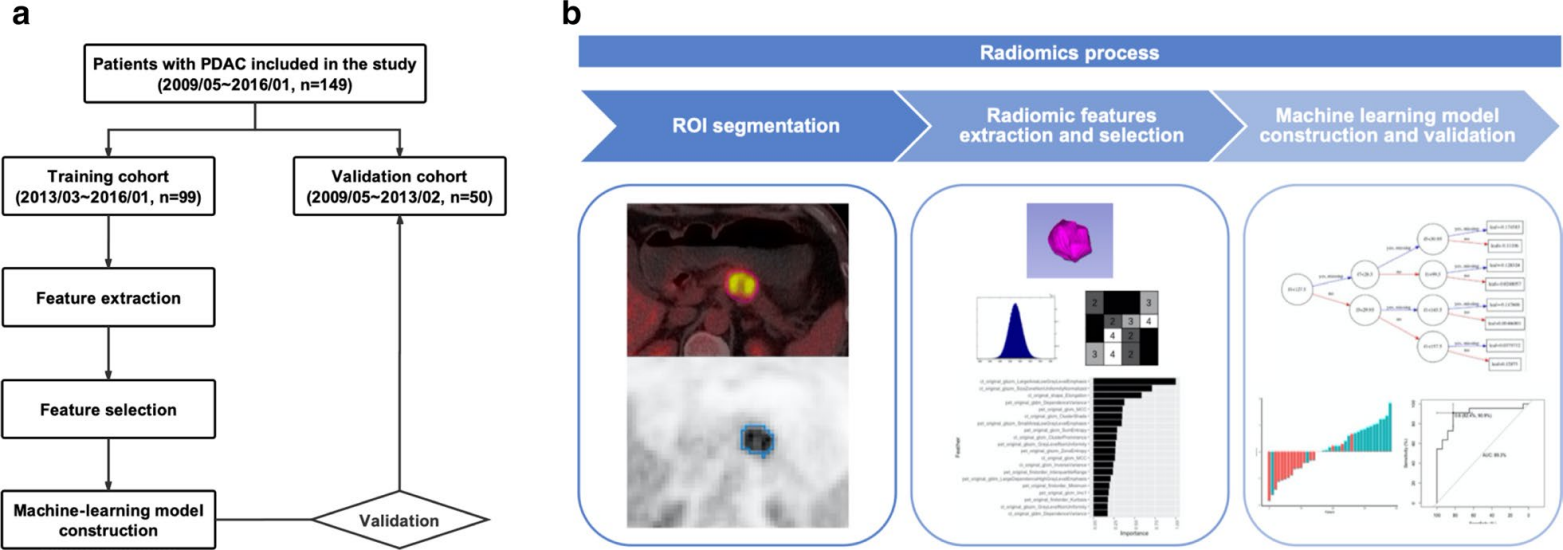
Analysis flowchart. **a** Study workflow. **b** Radiomics process

An overview of the study workflow is illustrated in Fig. 1, and the radiomics process is divided into five steps: region of interest (ROI) segmentation, radiomics feature extraction, feature selection, radiomics-based model construction, and model evaluation.

### Patients

This study was approved according to the guidelines for retrospective studies and rules of the institutional review board at Peking Union Medical College Hospital (PUMCH). Because of its retrospective nature, informed consent was waived, but all data were kept confidential. The inclusion criteria were as follows: (1) patients diagnosed with PDAC were confirmed pathologically, and (2) patients underwent preoperative ^18^F-FDG PET/CT scan within one month before surgery. And the exclusion criteria included: (1) patients had PDAC lesions which were too small to display clearly on PET/CT scan, or (2) patients had incomplete clinical and imaging data.

Based on the criteria of patient selection, a total of 149 patients diagnosed with PDAC between May 2009 and January 2016 at PUMCH comprised our study population. The patients were divided into two independent sets in the ratio of 2 to 1:99 patients performed PET/CT scan between March 2013 and January 2016 were taken as the training set, whereas the other 50 patients treated between May 2009 and February 2013 constituted the validation set.

Clinical characters (gender, age, and pathological grade of the tumor) were obtained from the hospital database. The lymphatic metastasis results on PET/CT images were interpreted by two nuclear medicine physicians who have 5–10 years of clinical experience in the diagnosis of abdominal diseases. The pathological grade (well-differentiated/grade 1, moderately differentiated/grade 2, and poorly differentiated/grade 3) of PDAC was classified according to the 2010 World Health Organization classification system [27]. Well-differentiated/grade 1 cases were classified as grade 1 group, whereas moderately differentiated/grade 2 and poorly differentiated/grade 3 cases were combined and classified as a single-grade 2/3 group since moderately to poorly differentiated tumor has overt and similar features of malignancy in pathology [28].

### PET/CT acquisition

The ^18^F-FDG PET/CT examination was performed from head to thigh using a Biograph 64 Truepoint TrueV scanner (Siemens Medical Solutions). All patients were instructed to avoid strenuous work or exercise for at least one day and fast for at least 4 h, and 0.15 mCi/kg of ^18^F-FDG was intravenously injected when blood glucose < 11.1 mmol/L. After injection, patients rested in a warm, dark room for 40–60 min. Then, a low-dose CT scan (CT scanning parameters: tube voltage 120 kV, slice thickness 3 mm, matrix size 512 × 512, and tube current adjusted through automatic modulation) for anatomical reference and attenuation correction was performed. The PET scan covering 5–6 bed positions (2 min per bed) immediately followed. PET images were reconstructed using iterative reconstruction (ordered subset expectation maximum, OSEM) and attenuation correction. A Gaussian smooth filter of 5 mm in full width at half-maximum was applied to the PET image (PET image parameters: resolution 4.07 × 4.07 mm, thickness 3 mm, and matrix size 200 × 200).

### ROI segmentation and radiomic features extraction

**Fig. 2:**
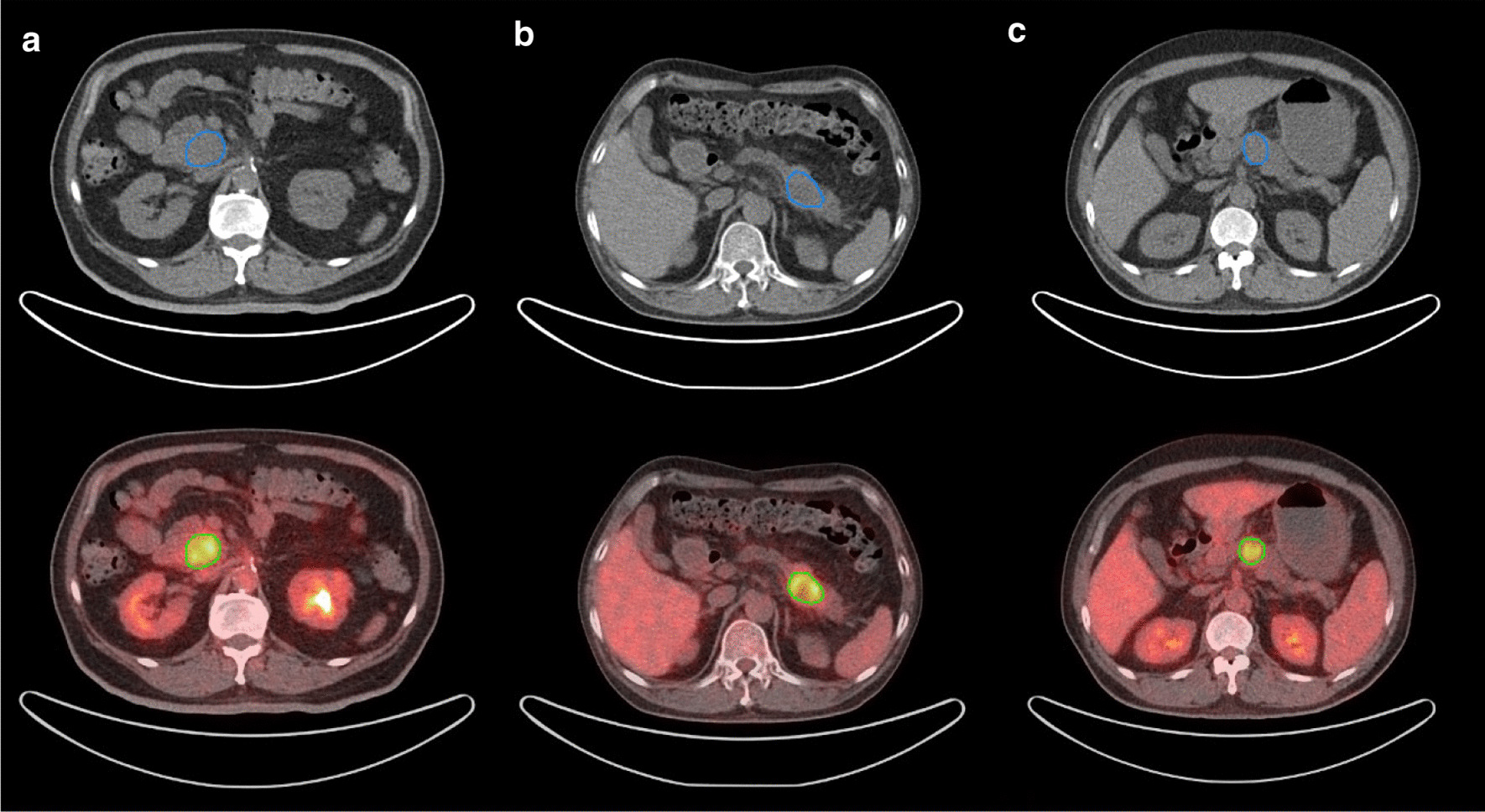
Examples of ROI segmentation in pancreatic ductal adenocarcinoma (PDAC) using ^18^F-FDG PET/CT images. One physician segmented tumor in blue along the edge of tumor (above), and the other physician segmented tumor in green to include voxels presenting SUV values greater than 50% of SUV_max_ (below). **a** The axial CT (above) and fusion (below) images of grade 3 (2010 World Health Organization classification system) PDAC in a 69-year-old man. The images showed a mass measuring about 3.7 × 3.4 × 2.8 cm with SUV_max_ of 3.7 in pancreatic head. **b** The axial images of grade 2 PDAC in a 57-year-old man. The image showed a mass with necrotic lesion in tail of pancreas, and SUV_max_ was 4.1. **c** The axial images of grade 1 PDAC in a 56-year-old man. The images showed a mass measuring about 2.7 × 2.7 × 2.8 cm with SUV_max_ of 3.0 in pancreatic body

The primary tumors were segmented by two nuclear medicine physicians using MIM software (version 6.6, MIM Software Inc.). The lesions were delineated automatically to include voxels presenting SUV values greater than 50% maximum SUV (SUV_max_) thresholds in the primary tumor. The MTV, TLG, mean SUV (SUV_mean_) of the primary tumor were calculated as the traditional quantitative volumetric parameters for PET imaging. The TLG was calculated as MTV multiplied by the SUV_mean_. For radiomics feature extraction, ROIs were delineated manually along the edge of primary tumor on CT images. All ROIs were then mapped to PET images. In order to map to the PET image, the ROI was resampled based on B-spline interpolation to ensure that it had the same pixel spacing as the PET image. Examples of ROI segmentation are shown in Fig. 2.

The radiomic features were extracted from both masked PET images and CT images using Pyradiomics implemented in Python [29]. Twenty-six shape-based features were obtained from the segmentation mask of CT images. About 3000 features, which consist of the features extracted from original and derived images (LoG with 5 sigma levels, 1 level of wavelet decompositions yielding 8 derived images, and images derived using square, square root, logarithm, and exponential filters), were obtained from PET and CT image. The fixed bin width (0.3 in SUV for PET images, 25 HU for CT images) was used to discretize the images.

### Machine learning and radiomics features selection

In this study, an extreme gradient boosting algorithm was used to build a robust machine learning-based classification. The classifier was constructed using XGBoost (version 0.81) in Python. XGBoost is a scalable end-to-end tree boosting system that is used widely by data scientists to achieve state-of-the-art results on many machine learning challenges [30]. It belongs to assembly algorithms that create and combine a set of individually weak classifiers to produce a robust estimator.

A model-based feature selection method was applied. The features were ranked based on the importance across all of the decision trees within the model. The importance is calculated for a single decision tree by the amount that each attribute split point improves the performance measure, weighted by the number of observations the node is responsible for. The average importance of which subsets split from the training set randomly was computed as the selection standard.

With the selected features, the machine learning-based classification was constructed by the training set. The area under the receiver-operator characteristic (ROC) curve (AUC) using the validation sets was calculated to assess the prediction accuracy of the classifier model. The radiomics signature of each patient was obtained from the score of the machine-learning classification algorithm before sigmoid transformation.

### Statistical analysis

Calculations were performed using SPSS software (version 26.0). Categorical variables are expressed as frequencies and percentages; continuous variables are expressed as mean ± standard deviation or median with interquartile range. The differences of patients’ characteristics between the training set and validation set as well as between the grade 1 group and grade 2/3 group were assessed using independent-sample *t* test, Mann–Whitney *U* test, and *χ*^2^ test. A *p* value of less than 0.001 was considered to indicate a statistically significant difference. The predictive accuracy of the machine learning model for predicting the histological differentiation of PDAC was evaluated using ROC analysis; the AUC was used as an index of accuracy.

## Results

### Clinical and biological characteristics of PDAC patients

In our study population, the training set and validation set had an even distribution in patients’ characteristics (Table 1). In the training set, 70 patients (70.7%) qualified as grade 2/3, and 29 (29.3%) as grade 1. The validation set proved insignificant distribution (*p* = 0.673), 37 patients (74.0%) qualified as grade 2/3, and 13 (26.0%) as grade 1. There were no significant differences regarding the other characteristics (gender, age, localization of tumor, PET/CT findings, and lymphatic metastasis) between the training set and validation set. The detailed distribution of patients’ characteristics in grade 1 and grade 2/3 group was summarized in Table 2. No significant group differences were observed between the grade 1 and grade 2/3 group both in the training set and validation set. This suggested that grade 1 and grade 2/3 PDAC could not be differentiated simply by PET/CT images and metabolic paraments.

**Table 1:** Characteristics of patients with PDAC in the training set and validation set

Characteristics	Training set (*n* = 99)	Validation set (*n* = 50)	*p* value
*Gender*			0.689
Male	54 (54.5)	29 (58.0)	
Female	45 (45.5)	21 (42.0)	
Age (years)	60.17 ± 10.48	62.62 ± 9.13	0.163
*Localization of tumor*			0.013
Head-neck	70 (70.7)	25 (50.0)	
Body-tail	29 (29.3)	25 (50.0)	
*Pathological grade*			0.673
Grade 1	29 (29.3)	13 (26.0)	
Grade 2/3	70 (70.7)	37 (74.0)	
*PET/CT findings*			
SUV_max_	4.62 (3.40, 6.96)	5.20 (3.72, 7.20)	0.408
SUV_mean_	2.98 (2.28, 4.28)	3.64 (2.48, 4.19)	0.359
MTV (ml)	11.17 (4.16, 19.62)	8.86 (2.39, 18.22)	0.533
TLG	33.82 (10.55, 66.65)	30.40 (9.71, 63.90)	0.816
*Lymphatic metastasis*			0.092
Present	58 (58.6)	22 (44.0)	
Absent	41 (41.4)	28 (56.0)	

Continuous data were expressed as mean ± standard deviation or median, with first and third quartile in parentheses. Categorical variables were expressed as numbers, with percentages in parentheses

**Table 2:** Characteristics of patients with PDAC in grade 1 and grade 2/3 group

Characteristics	Training set		*p* value	Validation set		*p* value
	Grade 1 (*n* = 29)	Grade 2/3 (*n* = 70)		Grade 1 (*n* = 13)	Grade 2/3 (*n* = 37)	
*Gender*			0.936			0.764
Male	16 (55.2)	38 (54.3)		8 (61.5)	21 (56.8)	
Female	13 (44.8)	32 (45.7)		5 (38.5)	16 (43.2)	
Age (years)	57.45 ± 9.69	61.30 ± 10.66	0.096	61.08 ± 9.33	63.16 ± 9.13	0.485
*Localization of tumor*			0.090			0.107
Head-neck	24 (82.8)	46 (65.7)		4 (30.8)	16 (43.2)	
Body-tail	5 (17.2)	24 (34.3)		9 (69.2)	21 (56.8)	
*PET/CT findings*						
SUV_max_	3.87 (2.61, 6.40)	4.65 (3.63, 7.22)	0.084	4.65 (3.72, 7.56)	5.30 (3.74, 7.19)	0.938
SUV_mean_	2.60 (2.03, 4.26)	3.11 (2.40, 4.31)	0.216	3.03 (2.68, 5.00)	3.81 (2.46, 4.14)	0.782
MTV (ml)	6.34 (2.21, 13.18)	12.42 (5.12, 20.02)	0.025	10.46 (3.23, 14.94)	8.54 (2.08, 20.00)	0.868
TLG	13.69 (6.04, 47.59)	39.72 (17.08, 67.81)	0.018	31.70 (12.54, 63.96)	29.19 (9.69, 63.72)	0.748
*Lymphatic metastasis*			0.651			0.264
Present	18 (62.1)	40 (57.1)		4 (30.8)	18 (48.6)	
Absent	11 (37.9)	30 (42.9)		9 (69.2)	19 (51.4)	

Continuous data were expressed as mean ± standard deviation or median, with first and third quartile in parentheses. Categorical variables were expressed as numbers, with percentages in parentheses

### Radiomics features selection and prediction model construction

The features were ranked by the score of boosted decision tree model. Finally, 6 PET predictive radiomics features (pet_wavelet-LLH_firstorder_Kurtosis, pet_wavelet-LLH_firstorder_Kurtosis, pet_wavelet-HHH_firstorder_Median, pet_lbp-2D_firstorder_Skewness, pet_gradient_glcm_Idmn, pet_wavelet-HLL_glcm_ClusterShade) and 6 CT features (ct_wavelet-HHH_glrlm_HighGrayLevelRunEmphasis, ct_wavelet-HLL_glcm_Imc1, ct_square_firstorder_Kurtosis, ct_lbp-3D-k_glcm_Correlation, ct_wavelet-LLL_glszm_LowGrayLevelZoneEmphasis, ct_wavelet-LLL_glszm_SizeZoneNonUniformity) were chosen to generate the radiomic signature. The naming convention of radiomics features is shown in Table 3. A model of XGBoost was constructed with the selected features using the training sets. The best parameters of the model were determined using grid search.

**Table 3:** Radiomics feature naming convention

Class	Abbreviation	Description
Modality	PET	Positron emission tomography
	CT	Computed tomography
Image Filter	Original	No filter applied
	Wavelet-HLH	Wavelet filtering
	Square	Takes the square of the image
	Gradient	gradient magnitude
	lbp-2D	Calculates and returns a local binary pattern applied in 2D
Matrix	firstorder	Voxel intensities within the image ROI through commonly used and basic metrics
	glcm	Gray-level co-occurrence matrix
	glrlm	Gray-level run length matrix
	glszm	Gray-level size zone
Index	Kurtosis	Kurtosis is a measure of the ‘peakedness’ of the distribution of values in the image ROI
	Median	The median gray-level intensity within the ROI
	Skewness	Skewness measures the asymmetry of the distribution of values about the mean value
	Idmn	IDMN (inverse difference moment normalized) is a measure of the local homogeneity of an image
	ClusterShade	Cluster shade is a measure of the skewness and uniformity of the GLCM
	Imc1	IMC1 assesses the correlation between the probability distributions of *i* and *j* in the GLCM
	HighGrayLevelRunEmphasis	HighGrayLevelRunEmphasis measures the distribution of the higher gray-level values in the GLRLM
	Correlation	Correlation is a value between 0 (uncorrelated) and 1 (perfectly correlated) showing the linear dependency of gray-level values to their respective voxels in the GLCM
	LowGrayLevelZoneEmphasis	LGLZE measures the distribution of lower gray-level size zones, with a higher value indicating a greater proportion of lower gray-level values and size zones in the image
	SizeZoneNonUniformity	SZN measures the variability of size zone volumes in the image, with a lower value indicating more homogeneity in size zone volumes

### Performance of prediction model

**Fig. 3:**
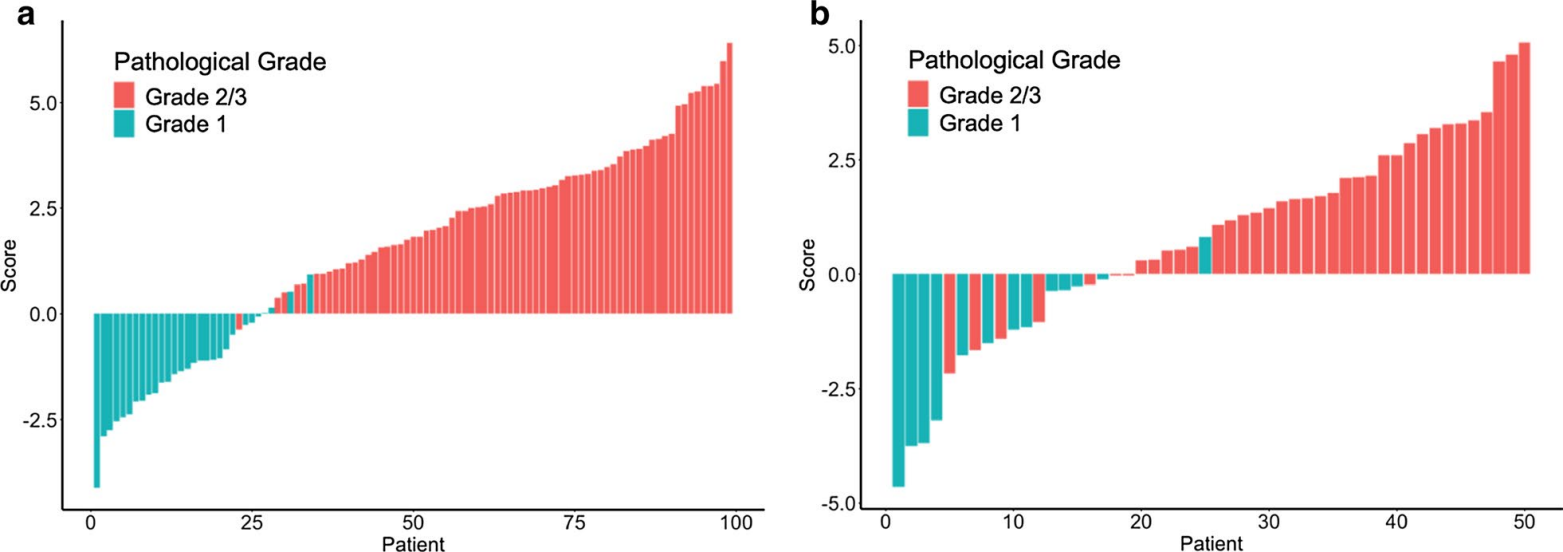
Radiomics score of each patient. **a** Training set. **b** Validation set

**Fig. 4:**
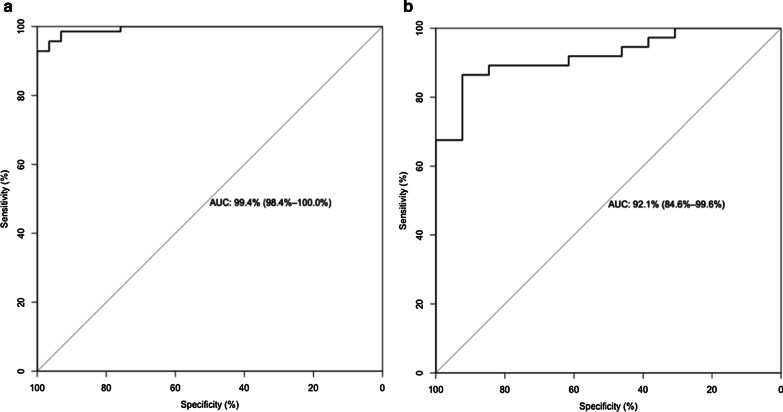
ROC curve. **a** Training set. **b** Validation set

Grade 2/3 patients generally displayed a higher radiomics score than grade 1 patients. There was a significant difference between the radiomics scores (median ± standard deviation) of the grade 2/3 and grade 1 patient groups in the training set [− 1.312 ± 1.128 vs. 2.784 ± 1.477, respectively, *p* < 0.001]; this difference was confirmed in the validation set [− 1.627 ± 1.684 vs. 1.599 ± 1.748, respectively, *p* < 0.001]. The radiomics score for each patient is shown in Fig. 3. The radiomics-based model achieved an AUC of 92.1% (95% CI 84.6–99.6%) in the validation set and 99.4% (95% CI 98.4–100%) in the training set (Fig. 4).

**Fig. 5:**
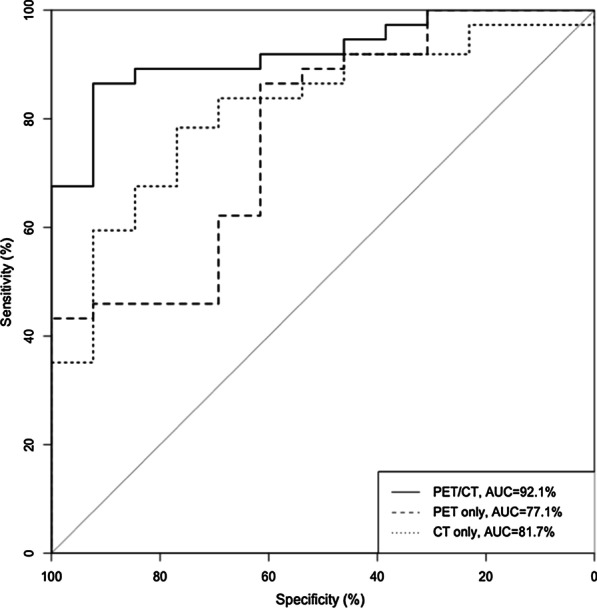
ROC curve of the difference modality radiomics-based model

Besides, we performed feature selection based on PET or CT image features only, and trained the prediction model separately, then compared the discrimination performance of them. The radiomics model based on PET and CT modality only achieved an AUC of 77.1% (95% CI 62.0–92.3%) and 81.7% (95% CI 69.0–94.5%) in the validation set, respectively. Figure 5 demonstrates the ROC curve of the model with PET/CT, PET, and CT modality in the validation set.

## Discussion

The purpose of this retrospective study is to investigate the value of PET/CT radiomics for predicting the risk assessment of PDAC. In this work, we present a model based on 12 radiomics features found to be effective for the classification, which could stratify PDAC patients into grade 1 and grade 2/3 groups with AUC of 0.994 in the training set and 0.921 in the validation set. The results suggested that PET/CT radiomics with machine learning may contribute to the preoperative risk assessment of PDAC patients.

Differentiation grade is an important biologic feature of malignant tumors. Although pathological examination is a necessary exam to determine the histological classification of the tumor, radiomics might have an increasing clinical role, as it provides an atraumatic in vivo and simple method to characterize the lesions. Some scientists have explored the value of PET-radiomics in tumor classification. Hyun et al. successfully identified the histological subtypes of lung cancer using a machine-learning algorithm with PET-based radiomic features [31]. Also, Han et al. showed that machine learning/deep learning algorithms could help radiologists to differentiate the histological subtypes of non-small cell lung cancer via PET/CT images [32]. Zhang et al. confirmed that the quantified radiomics method could aid the noninvasive differentiation of autoimmune pancreatitis and PDAC in ^18^F-FDG PET/CT images [26]. Similar to the above-mentioned studies, current one also supports the conclusion that PET/CT texture analysis is a promising method for predicting the differentiation or subtype of the tumor.

Several studies have evaluated the potential value of radiomics for predicting pathology of pancreatic tumors. Attiyeh et al. present a multivariate model of quantitative imaging features and clinical variables that predicts low and high risk in intraductal papillary mucinous neoplasms [33]. Kaissis et al. developed a machine learning algorithm from diffusion-weighted imaging-derived radiomic features, which has the potential in PDAC preoperative subtyping [24]. Furthermore, Liang et al. developed a nomogram model combing radiomics features and clinical characteristics, and it showed the best performance in differentiating grade 1 and grade 2/3 pancreatic neuroendocrine tumors [10]. Recently, Qiu et al. found that there were significant differences between low-grade PDAC (well-differentiated and moderately differentiated) and high-grade PDAC (poorly differentiated) regarding 18 CT texture features [34]. These studies are mostly based on CT and MRI modality. We collected PET/CT images as the image modality in this study. CT or MRI reflects mainly the anatomical structure of tumors, while PET/CT can be used to explore intratumoral heterogeneity in both anatomical and functional dimensions [35]. In our study, the ^18^F-FDG PET/CT-based texture analysis demonstrated excellent performance in identifying the pathological grade of PDAC.

The current study showed that CT radiomics features are better in predicting the pathologic grade of PDAC than PET features. This finding is as expected since PET images has lower resolution than CT images, and previous studies have indicated that the performance of texture analysis is better in the analysis of CT images than PET images [36]. Kirienko et al. found that the added value of PET appears limited when comparing the AUC of radiomic signature among CT, PET, and PET + CT in the prediction of disease-free survival of non-small cell lung cancer [37]. However, there are very limited literatures on the role of radiomics of both PET and CT images in pancreatic tumor. Our study shows that the model using both the PET and CT features has an advantage compared to using either PET or CT features alone in predicting the pathologic grade of PDAC. Also it is worth noting that texture analysis of CT images was performed using the CT component of the PET/CT instead of diagnostic CT images, so evaluating the predictive performance of PET/CT and diagnostic CT is an interesting and valuable research direction. Besides, we attempted to divide patients into training and validation set in another chronological order in the ratio of 2 to 1, but the distribution between two sets was not even (Additional file 1: Fig. 1 and Table 1), with the lymphatic metastasis demonstrated significant difference between the training and validation set (*p* < 0.001). Thus, we did not adopt this grouping method. In the studies of radiomics, validation is an important part in assessing the performance model. It is necessary to fully consider the influence of training data and validation data. Valid models should exhibit statistical consistency between the training and validation sets [21]. Furthermore, externally validated model has more credibility than internally validated model, because data obtained with the former approach are considered more independent [38].

It is interesting to note that in this study, features extracted from wavelet filtered images played a significant role in the prediction model. Wavelet transform can decompose images into low- and/or high-frequency components at different scales, and the intensity and textural features exacted from wavelet decompositions of the original image can represent different frequency ranges within the tumor volume. Previous studies have shown that wavelet transform-based radiomic features can serve as independent predictive or prognostic factors [39, 40], and are correlated with histological classification results and/or genes mutation status [41, 42]. Our study also suggests the value of wavelet features in identifying the pathological grade of PDAC.

As a retrospective study, a limitation of our study is that the number of enrolled patients was relatively small. Because of the sample number of poorly differentiated cases was small, grade 2/3 PDAC group was not further separated into moderately and poorly differentiated cases. Also, the present study depends on the pathological diagnosis; thus, our cohort was restricted to resected patients, and selection bias may exist implicitly. In the future, we will expand our patient cohort and validate the ability of this prediction model to separate well, moderately, and poorly differentiated patients in a prospective study.

In conclusion, this model based on PET/CT radiomics and machine learning can predict the histological differentiation of PDAC effectively and preoperatively. Before it can be used in clinical practice, prospective studies on larger patient cohorts are required to validate its utility further. As the development and standardization of PET/CT radiomics, it could be a promising preoperative, noninvasive, precise, and simple approach that can assist physicians in evaluating the risk of patients with PDAC individually, and thus achieving a personalized treatment and a better clinical outcome.

## Supplementary Information

**Additional file 1: Supplement Fig. 1:** Analysis flowchart. **Supplement Table 1:** Characteristics of patients with PDAC in the training set and validation set.

## Data Availability

Not applicable.
