# Supplementary material for: Preoperative prediction of pathological grade in pancreatic ductal adenocarcinoma based on 18F-FDG PET/CT radiomics
Source: EJNMMI Res. 2021 Feb 25;11:19. doi: 10.1186/s13550-021-00760-3 (PMC7907291; doi:10.1186/s13550-021-00760-3)
Supplement: Supplementary file 1 — Additional file 1: Supplement Fig. 1: Analysis flowchart. Supplement Table 1: Characteristics of patients with PDAC in the training set and validation set. [file 13550_2021_760_MOESM1_ESM.docx]

**Supplementary Material**

We attempted to divide patients into training and validation set in another chronological order in the ratio of 2 to 1. 99 patients performed PET/CT scan between May 2009 and August 2014 were taken as the training set, whereas the other 50 patients treated between September 2014 and January 2016 constituted the validation set (Supplement Fig. 1). But the distribution between two sets was not even (Supplement Table 1), with the lymphatic metastasis demonstrated significant difference between the training and validation set (*p*<0.001). We did not adopt this grouping method at last.


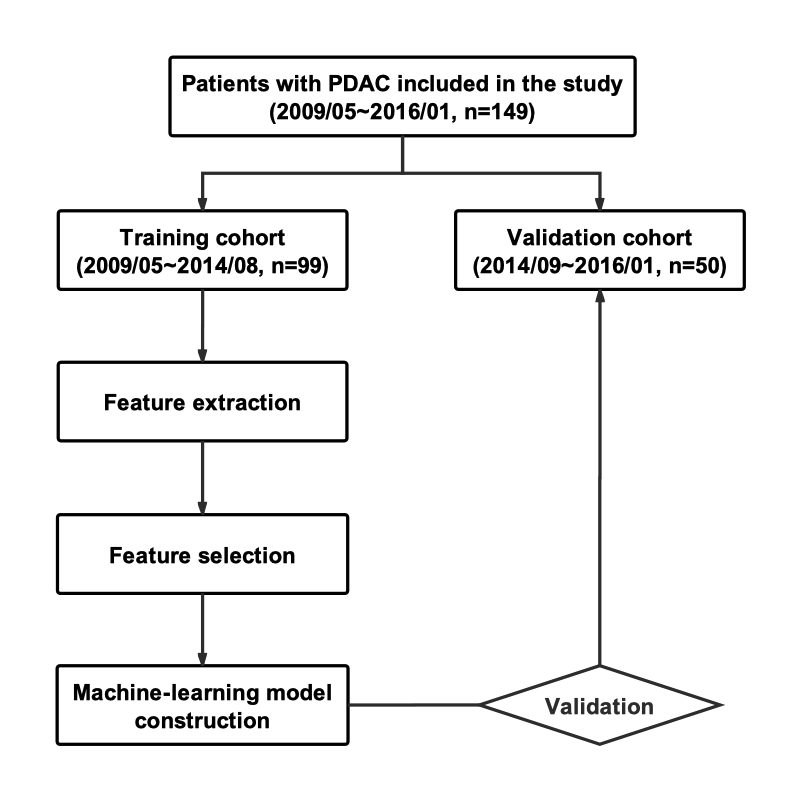


**Supplement Fig. 1** Analysis flowchart.

**Supplement Table 1** Characteristics of patients with PDAC in the training set and validation set

| Characteristics | Training set (n=99) | Validation set (n=50) | *p* value |
| --- | --- | --- | --- |
| Gender  Male  Female | 60 (60.6)  39 (39.4) | 23 (46.0)  27 (54.0) | 0.090 |
| Age (years) | 61.03±9.06 | 60.92±11.97 | 0.950 |
| Localization of tumor  Head-neck  Body-tail | 57 (57.6)  42 (42.4) | 38 (76.0)  12 (24.0) | 0.027 |
| Pathological grade  Grade 1  Grade 2/3  PET/CT findings  SUV_max_  SUV_mean_  MTV (ml)  TLG  Lymphatic metastasis | 33 (33.3)  66 (66.7)  5.09 (3.62, 7.17)  3.35 (2.48, 4.29)  8.54 (3.23, 18.08)  29.19 (9.74, 63.84) | 9 (18.0)  41 (82.0)  4.32 (3.18, 6.85)  2.92 (2.11, 4.09)  12.96 (5.14, 20.59)  37.61 (13.64, 67.59) | 0.049  0.376  0.137  0.118  0.469  <0.001* |
| Present  Absent | 21 (21.2)  78 (78.8) | 25 (50.0)  25 (50.0) |  |

*Statistical significance of lymphatic metastasis on PET/CT images between training and validation set.

Continuous data were expressed as mean ± standard deviation or median, with first and third quartile in parentheses. Categorical variables were expressed as numbers, with percentages in parentheses.
